# Aspirin resistance in patients with acute coronary events: risk factors and prevalence as determined by whole blood multiple electrode aggregometry

**DOI:** 10.12669/pjms.296.3608

**Published:** 2013

**Authors:** O. Ibrahim, O. Maskon, Noor Darinah, A. A. Raymond, M. M. Rahman

**Affiliations:** 1 O. Ibrahim, Department of Medicine, University Kebangsaan Malaysia Medical Centre, Cheras 56000, Kuala Lumpur, Malaysia.; 2 O. Maskon, Department of Medicine, University Kebangsaan Malaysia Medical Centre, Cheras 56000, Kuala Lumpur, Malaysia.; 3 Noor Darinah, Department of Medicine, University Kebangsaan Malaysia Medical Centre, Cheras 56000, Kuala Lumpur, Malaysia.; 4 A A Raymond, Department of Medicine, University Kebangsaan Malaysia Medical Centre, Cheras 56000, Kuala Lumpur, Malaysia.; 5 M.M. Rahman, Department of Medical Microbiology & Immunology,

**Keywords:** Aspirin resistance, aspirin responsiveness, first-ever coronary event, recurrent coronary event, Multiplate® platelet analyser

## Abstract

***Objectives:*** To determine the prevalence of aspirin resistance and associated risk factors based on biochemical parameters using whole blood multiple electrode aggregometry.

***Methods:***The study was conducted at the outpatients cardiology clinic of the Universiti Kebangsaan Malaysia Medical Centre (UKMMC) from August 2011 to February 2012.

Subjects on aspirin therapy were divided into two groups; first-ever coronary event and recurrent coronary event. Aspirin resistance was measured by a Multiplate^®^ platelet analyser.

***Results:*** A total of 74 patients (63 male, 11 female), with a mean age of 57.93 ± 74.1years were enrolled in the study. The patients were divided into two groups –first-ever coronary event group (n=52) and recurrent coronary event group (n=22). Aspirin resistance was observed in 12 out of 74 (16%) of the study patients, which consisted of 11 patients from the first-ever coronary event group and one patient from the recurrent coronary event group. There were significant correlations between aspirin resistance and age (r = -0.627; p = 0.029), total cholesterol (r = 0.608; p = 0.036) and LDL (r = 0.694; p = 0.012). LDL was the main predictor for area under the curve (AUC) for aspirin resistance. However, there was no association between aspirin resistance and cardiovascular events in both groups in this study.

***Conclusions:*** Aspirin resistance was observed in 16% of the study population. LDL was the major predictor of aspirin resistance. No association was found in the study between aspirin resistance with recurrent coronary events.

## INTRODUCTION

Antiplatelet therapy with aspirin (ASA) is one of the cornerstones in acute coronary syndrome. Previous meta-analyses of randomised trials have shown that antiplatelet therapy prevents serious vascular events, arterial occlusion and venous thromboembolism in patients at high risk of occlusive vascular events.^   1 ^

Aspirin works by inhibiting the prostaglandin-producing enzyme cyclooxygenase, which converts arachidonic acid into prostaglandin. Several previous studies have shown biochemical aspirin resistance and its association with clinical outcomes.^2^^-^^4^

In this study, platelet function analysis was performed using the Multiplate® platelet analyser (Dynabyte, Munich, Germany), a novel whole blood impedance aggregometer. It is one of the most widely applied platelet aggregometers in Europe. It has been used to study the effects of aspirin,^5^^,^^6^ non-opioid analgesics,^7^ clopidogrel,^8^^,^^9^ andanticoagulants^10^ on platelet aggregation. We have previously evaluated clopidogrel and aspirin resistance in different groups of patients with coronary events.^11^ However, studies comparing aspirin resistance in patients with the first-ever coronary and recurrent coronary events are scanty.

Therefore, the present study was undertaken to determine the prevalence of aspirin resistance in patients with first-ever vs. recurrent coronary events and its associated risk factors based on biochemical parameters using whole blood multiple electrode aggregometry.

## METHODS

***Patient population: ***This study was conducted at the Universiti Kebangsaan Malaysia Medical Centre (UKMMC) between August 2011 and February 2012. The research was approved by the Ethics Committee of the UKMMC (FF-244-2011).

**Definition of groups used in the study:**

1. First-ever coronary event group; patients on aspirin who had their first-ever coronary event.
2. Recurrent coronary event group; patients on aspirin who had had previous coronary artery disease (CAD) and at least one previous coronary event.

All patients aged 18 to 65 years diagnosed with stable coronary artery disease (CAD) who were on Aspirin (ASA) 150mg daily, and who had either their first ever or recurrent coronary event were included in this study. These patients were followed up for a period of six months. Patients who had ASA allergy or could not tolerate it due to gastric disease such as peptic ulcer disease (PUD), were excluded from the study.

***Patient assessment and blood sample collection: ***Informed consent was obtained from the patients before taking a full history and performing the physical examination. Blood was withdrawn for platelet aggregation evaluation using a Multiplate® platelet analyzer. Blood was also sent for full blood count Fasting Serum Lipid (FSL). Full Blood Count(FBC), renal profile (RP), HbA1c and lipid profile.

***Method of performing the test using Multiplate® platelet analyzer:*** The test was performed as per method described by Ibrahim et al.^11^ Briefly after positioning and connecting the test cells; 300µl of the saline and 300µl of whole blood sample (hirudinised) was pipetted into each cell. Results took six minutes to be ready and the aggregation results were shown in AU units, velocity and area under the curve (AUC). We used a cut of value of > 300 AUC^*^min as set by themanufacturer^5^^,^^12^ to establish the presence of aspirin resistance.

***Statistical analysis: ***Data were analyzed using the SPSS version 15.0 statistical package. Quantitative and qualitative demographic characteristics were summarized and data were tabulated and tested for normality with the Shapiro-Wilk test because the sample was below 100. All statistical tests were carried out at a significant level of p value < 0.05. Data were expressed as mean +/- standard deviation (SD), median (95%CI) and inter-quartile range (IQR) or proportion or percentage for all data.

## RESULTS

Patients’ demography: Seventy four patients were recruited from the coronary care unit and medical wards in which fifty two patients fell under the first-ever coronary event group and twenty two under the recurrent coronary event group.The patients had the following demographic and clinical features (Table-I).

**Table-I:** Demographic and clinical characteristics of the patients

*Characteristic*	*First-ever coronary event group* *(n=52)*	*Recurrent* *coronary event group* *(n=22)*	*P value*
Mean-age-Years	57.04 ± 7.49^a^	59.59 ± 6.45^a^	NS
Male sex-no (%)	47 (90%)	16 (73%)	NS
BMI (kg/m^2^)	28.11 ± 5.01^a^	25.39 ± 6.33^a^	NS
SBP	133.50 (24.25)^b^	129.50 (18.75)^b^	NS
DBP	77.50 (15.00)^b^	75.00 (24.25)^b^	NS
DM-no (%)	26 (50%)	7 (32%)	NS
Active Smoker-no (%)	9 (17%)	2 (9%)	NS
Previous CAD(n)	52	22	
Unstable angina	14 (27%)	5 (23%)	NS
NSTEMI	16 (31%)	13 (59%)	NS
STEMI	22 (42%)	4 (18%)	NS
Duration on aspirin (year)	5.00 (7.0 )^b^	7.00 (6.5)^b^	NS

a = Mean ± (SD; standard deviation); b = Median (IQR; interquartile range) SBP,Systolic Blood Pressure; n= Number

DBP, Diastolic Blood Pressure; BMI= Body Mass Index, DM (Diabetes Mellitus),

NSTEMI (non-ST elevation myocardial infarct), STEMI (ST elevation myocardial infarct),NS= Not Significant.

Aspirin resistance was found in 12 out of 74 patients (16%), from the first-ever coronary event group and one from the recurrent event group. The results for AUC, aggregation and velocity were not significantly different between both study groups (Table-II).

**Table-II:** AUC, Aggregation and Velocity results of the “First-EverCoronary Event” Group and “Recurrence Coronary Event” Group

*Parameter*	*First-ever* *Coronary event group (n=52)*	*Recurrent* *Coronary event group (n=22)*	*p value*
Multiplate® platelet analyzer result			
AUC (AU^*^min)	197.15 ± 310.32^a^	146.27 ± 183.48^a^	0.385
Aggregation (AU)	37.74 ± 53.87^a^	29.62 ± 30.05^a^	0.412
Velocity (AU/Min)	6.28 ± 6.82^a^	6.40 ± 8.31^a^	0.955

a = Mean ± SD (standard deviation); b = Median (IQR; interquartile range)

Aspirin resistant patients had significantly higher levels of total cholesterol and LDL compared to aspirin responsive patients (p=0.021 and 0.027,respectively) (Table-111). No other significant differences between the two groups were observed.

**Table-III:** Biochemical markers of the “First-EverCoronary Event” Group and “Recurrence Coronary Event’” Group

**Characteristic**	**First-ever coronary event group** **(n=52)**	**Recurrent** **coronary event group** **(n=22)**	**P value**
Full blood count			
Haemoglobin (g/dL)	12.63±1.33^a^	13.99±1.81^a^	0.031
Total White Cell Count(x10^9^/L)	8.54± 2.22^a^	± 2.81^a^	NS
Platelet (x10^9^/L)	279.50 (117.00)^b^	229.50(75.50)^b^	0.049
Lipid profile			
Total Cholesterol (mmol/L)	5.29 (1.54)^b^	4.41 (1.59)^b^	0.021
TG (mmol/L)	1.60 (0.60)^b^	(0.90)^b^	NS
LDL (mmol/L)	3.74 (1.77)^b^	2.66 (1.41)^b^	0.027
HDL (mmol/L)	1.04 (0.78)^b^	1.21 (0.80)^b^	NS
Renal profile			
Sodium (mmol/L)	139.00 (3.50)^b^	139.00(3.00)^b^	NS
Potassium (mmol/L)	4.35 (0.65)^b^	4.30( 0.50)^b^	NS
Urea (mmol/L)	4.50 (1.55)^b^	1.95)^b^	NS
Creatinine (umol/L)	82.50 (29.25)^b^	92.00(33.50)^b^	NS
Creatinine Clearance (mL/min/1.73m^2^)	82.00 (29.00)^b^	72.00 (27.00)^b^	NS
HbA1c (%)	6.50 (2.98)^b^	5.95 (1.53)^b^	NS
Fasting Blood Glucose(mmol/L)	6.20(4.28)^b^	6.00(2.63)^b^	NS

Using the Spearman Correlation Test, there was negative correlation between age and area under curve (AUC) of biochemical aspirin resistance (r = - 0.627; p = 0.029). Total cholesterol and low-density lipoprotein (LDL) had positive correlation with AUC of aspirin resistance (r=0.608; p = 0.036 and r = 0.694; p = 0.012, respectively).

There was no association between biochemical aspirin resistance and cardiovascular events (Table IV).

**Table-IV:** Contingency table of biochemical markers in aspirin responsive study population (n=74).

*Group*	*First-evercoronary event group (n=52)*	*Recurrent coronary event group (n=22)*	Chi-Square test*
*Biochemical*			
Aspirin responsive	41 (55%)	21 (29%)	X^2^= 2.634 and p= 0.105
Aspirin resistance	11 (15%)	1(1%)	

*There was no association between aspirin responsive or aspirin resistance with groups of first-ever coronary event or recurrentcoronary event.

## DISCUSSION

In this prospective study we determined the prevalence of, and risk factors for aspirin resistance in patients who had a first-ever coronary event versus those who had a recurrent coronary event, and who were given standard dose aspirin (150mg daily).

The mean age of our study population was 57.93 years and the male gender constituted 90% and 73% of the patients in the first and second groups, respectively. The mean blood pressure in both groups was within the normal range. In this study, the prevalence of aspirin resistance was 16% as determined by the Multiplate® platelet analyzer. Previous studies^13^^-^^15^ showed that the prevalence of aspirin resistance varied between 0.4%^16^ and 56.8%^17^ in patients with adverse recurrent cardiovascular events. The variations might be due to the different methods used by the researchers for the evaluation. Thus, until now it is still unclear which parameters are the most accurate in estimating aspirin resistance.

Among the 12 patients with aspirin resistance, LDL was found to be the main predictor for AUC and had a positive correlation with aspirin resistance as well as total cholesterol values while ‘age’ had negative correlation in the twelve patients with biochemical aspirin resistance. We can conclude that the risk factors for aspirin resistance in these 12 patients could be due to high LDL and total cholesterol.Other studies reported that high LDL^18^^, ^^19^was due to reduced response of platelets towards aspirin. The significance of the negative correlation between age and aspirin resistance in the study patients with biochemical aspirin resistance has not been reported before. Previous study reported that there was a trend toward aspirin resistance with increasing age.^12^

Our study did not demonstrate any association between aspirin resistance and other risk factors such as body mass index (BMI), blood pressure, full blood count, renal profile, HDL, triglyceride and creatinine clearance. This finding might be due to the limited number of sample size and short duration of study

In this study, there was no association between either aspirin responsiveness or aspirin resistance with the first-ever coronary event or recurrent coronary event. This is in keeping with two previous prospective studies reported by Christiaens et al.^18^^,^^20^ but not with a few other studies^2^^-^^4^^,^^20^ The differences in study duration and sample size between these studies may contribute to the differences in study outcome.
